# Pseudodrusen in the Fellow Eye of Patients with Unilateral Neovascular Age-Related Macular Degeneration: A Meta-Analysis

**DOI:** 10.1371/journal.pone.0149030

**Published:** 2016-02-19

**Authors:** Qiang Zhou, James Shaffer, Gui-shuang Ying

**Affiliations:** 1 Department of Ophthalmology, Beijing Chaoyang Hospital, Capital Medical University, Beijing, P. R. China; 2 Department of Ophthalmology, Scheie Eye Institute, Perelman School of Medicine, University of Pennsylvania, Philadelphia, Pennsylvania, United States of America; Tufts University, UNITED STATES

## Abstract

**Importance:**

The fellow eye of patients with unilateral neovascular age-related degeneration (nAMD) is at increased risk of developing late AMD. Several cohort studies have evaluated the prevalence of pseudodrusen and the association between pseudodrusen and late AMD in the fellow eye of patients with unilateral nAMD. However, these studies have limited sample sizes and their results are inconsistent.

**Objective:**

To evaluate the prevalence rate of pseudodrusen, and the association between pseudodrusen and incidence of late AMD (nAMD and geographic atrophy (GA)) in the fellow eye of patients with unilateral nAMD.

**Data Sources:**

The PubMed, EMBASE, Web of Science, and Cochrane Library databases were searched up to July 2015, as well as other systematic reviews.

**Study Selection:**

All cohort studies for pseudodrusen with late AMD in the fellow eye of patients with unilateral nAMD.

**Data Extraction and Synthesis:**

The numbers of patients with and without pseudodrusen at baseline and the numbers of incident nAMD and GA during follow up among patients with and without pseudodrusen were independently extracted by 2 authors. The results were pooled using random-effects meta-analysis. Heterogeneity was assessed using the I^2^ test.

**Main Outcome Measures:**

Prevalence rate of pseudodrusen, risk ratios (RRs) and their 95% confidence intervals (95% CIs) for associations between pseudodrusen and the incidence of nAMD and GA in the fellow eye.

**Results:**

Five cohort studies (N = 677 patients) from 8 countries across 4 continents were included. The pooled prevalence rate of pseudodrusen in the fellow eye was 48.1% (95% Cl: 36.7–59.5%, I^2^ = 87%). Pseudodrusen were associated with an increased risk of nAMD (RR = 1.54, 95% Cl: 1.10–2.16, I^2^ = 42%), GA (RR = 4.70, 95% Cl: 1.22–18.1, I^2^ = 64%), and late AMD (RR = 2.03, 95% Cl: 1.35–3.06, I^2^ = 60%).

**Conclusions:**

For patients with unilateral nAMD, pseudodrusen were present in about half of the fellow eyes. The presence of pseudodrusen was associated with a 1.5 times higher risk of developing nAMD, a 4.7 times higher risk of developing GA, and a 2 times higher risk of developing late AMD. Pseudodrusen should be considered in evaluating the risk of late AMD development; however, due to considerable heterogeneity across these studies, a larger study is needed to validate these findings.

## Introduction

Age-related macular degeneration (AMD) is the leading cause of blindness among the elderly in Western countries and is the third leading cause of blindness in the world.[1, 2] Many risk factors have been reported for AMD progression to vision-threatening late-stage AMD, and drusen is one of the typical harbingers.[3] In 1990, Mimoun et al. first reported a special type of drusen in the macula of patients with AMD, which is described as “pseudodrusen visible en lumiere bleu” because it is visible in blue light and did not appear hyperfluorescent on fluorescein angiography.[4] Later, Arnold et al named them as “reticular pseudodrusen”[5], and other terms like “reticular drusen” and “reticular macular disease/lesions” were also used to describe this special type of drusen by various authors.[6, 7] Recent studies have used spectral domain optical coherence tomography (SD-OCT) and have demonstrated that reticular pseudodrusen are subretinal drusenoid deposits.[8–11] The subretinal location has been confirmed with histology and adaptive optics scanning laser ophthalmoscopy.[12–14]

The Beaver Dam Eye Study and the Blue Mountains Eye Study reported 4.0% and 6.6% 15-year pseudodrusen incidence rates based on the grading of standard color fundus photography and found that the proportion of eyes with pseudodrusen that progressed to late AMD within 5 years was four-fold to six-fold higher than eyes without pseudodrusen but with other early AMD features.[15–17] Several clinical studies also demonstrated a strong association between pseudodrusen and the development of late AMD. [5, 7, 9, 18–20] Evaluating the association between pseudodrusen and late AMD development in the fellow eye is clinically important because patients with unilateral neovascular AMD have a high risk of developing late AMD in the fellow eye, and this risk may be significantly increased by the presence of pseudodrusen. In recent years, a few studies have investigated the presence of pseudodrusen in the fellow eye of patients with unilateral nAMD; however, these studies are limited in their sample size (ranging from 20 to 271 patients), and only a small number of patients developed nAMD or geographic atrophy (GA). The statistical significance and magnitude of the association between pseudodrusen and late AMD varied substantially (the risk ratio ranged from 1.8 to 9.7), and the conclusions on the relative relationship with nAMD and GA from these studies are inconsistent. Therefore, we conducted a meta-analysis to evaluate the prevalence rate of pseudodrusen and the associations between pseudodrusen and incidence of nAMD, GA and late AMD in the fellow eye of patients with unilateral nAMD.

## Methods

This meta-analysis was conducted following the Meta-analysis of Observational Studies in Epidemiology (MOOSE) statement.[21]

### Data Sources and Searches

The PubMed, EMBASE, Web of Science, and Cochrane Library databases were searched up to July 2015 using combinations of the following search terms: (pseudodrusen OR reticular drusen OR reticular macular disease OR subretinal drusenoid deposits) AND fellow eye. No language restrictions were imposed.

The titles and abstracts of the retrieved articles were reviewed to exclude any clearly irrelevant studies. The full texts of the remaining articles were then reviewed to determine whether they contained any information on the prevalence of pseudodrusen and the association with the incidence of late AMD (either nAMD or GA). To find all relevant studies, the reference lists of systematic reviews on pseudodrusen,[22–25] as well as the reference lists of each included study, were also comprehensively checked.

### Study Selection and Data Extraction

The inclusion criteria for this meta-analysis were: (1) the study patients had unilateral nAMD; (2) the presence of pseudodrusen was evaluated in the fellow eye using at least two imaging modalities; and (3) the study outcomes were incidence of either nAMD or GA. We (QZ and GSY) independently assessed the eligibility of studies and extracted data from each eligible study, and the discrepancies were discussed to reach agreement.

The study characteristics and results from eligible studies were extracted, including the name of the first author, the year of publication, the sample size, the country of study, the mean age of participants, the number of years of follow-up, the imaging modalities used for detecting pseudodrusen, the prevalence of pseudodrusen at baseline, and the incidence of nAMD and GA. We contacted the corresponding authors of retrieved articles if additional data were needed.

### Statistical Analysis

Open Meta-Analyst software[26] (version for windows 8, downloaded from http://www.cebm.brown.edu/open_meta/download.html) was used for the statistical analysis. Random-effects models were used to estimate the pooled prevalence rate of pseudodrusen and to estimate the risk ratios (RRs) and their 95% confidence intervals (95% CIs) for the associations of pseudodrusen with the incidence of nAMD, GA and late AMD. The pooled RR and its 95% CI for CNV and GA were not adjusted by other covariates, because the covariates-adjusted RRs for CNV and GA were not reported in most studies. However, for late AMD, the pooled covariate-adjusted RR and its 95% CI were calculated from the individual covariates-adjusted RR (adjusted by age, gender, drusen and pigmentation changes) from 3 studies.[27–29] The percentage of total variation in the effect estimates across included studies due to heterogeneity was assessed using the I^2^ statistic.[30] The I^2^ ranges between 0% and 100% (0% represents no heterogeneity), and higher values represent larger heterogeneity. To account for the various length of follow-up across studies, we also calculated the annual incidence rate of nAMD, GA and late AMD by using the person years method. A sensitivity analysis, removing each study one at a time, was performed to confirm the robustness of our findings.

## Results

**Fig 1:**
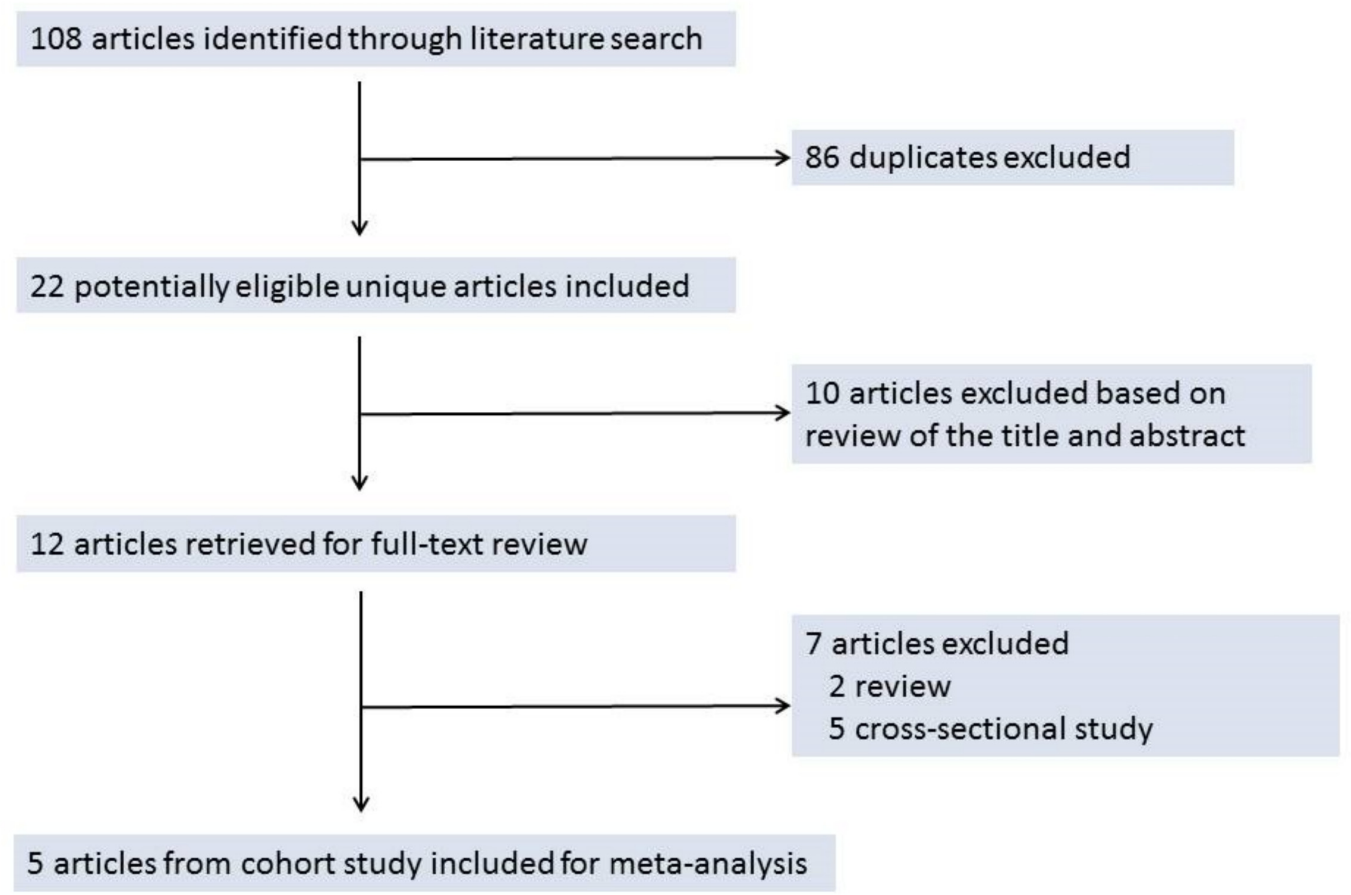
The Selection of Studies for the Meta-analysis.

As summarized in Fig 1, 108 articles were identified from the databases (23 from PubMed, 9 from Cochrane Library database, 44 from Web of Science, and 32 from Embase). After removing 86 duplicates, 22 unique articles were considered to be of potential relevance. Based on the review of the abstract, 10 of these 22 articles were deemed as irrelevant and the remaining 12 articles were retrieved for full-text review, 5 of which met our inclusion criteria and were include in the meta-analysis.[27–29, 31, 32]

The characteristics of the 5 included studies are summarized in Table 1. All five studies were conducted in the past 5 years, and represent patients from 8 countries across 4 continents, with two studies from Asia (Japan, Korea), four from Europe (France, Italy, Portugal, and United Kingdom), one from Australia and one from the United States. The combined total sample size was 677 patients with individual study sizes ranging from 20 to 271 patients. The mean age ranged from 74 to 83 years. The mean length of follow-up ranged from 2 to 4 years.

**Table 1:** Characteristics of the Studies Included in the Meta-analysis.

Study (first author, journal, year)	Country of study participants	Study design	Sample Size	Mean age (SD) in years	Women/men	Frequency of follow-up	Years of follow-up	Image modalities for detecting pseudodrusen
**Pumariega NM, *Ophthalmology*, 2011[29]**	France	Prospective, single-center, clinic-based	271	74(6.7)	171/100	At 6 months and at the end of years 1, 2, and 3	3	BC, BLP, CFP, FA, RF
**Sawa M, *Retina*, 2014[32]**	Japan	Retrospective, single-center, clinic-based	20	83 (NA)	11/9	Every 1–3 months	4	BC, RF, SD-OCT
**Finger RP, *Ophthalmology*, 2014[27]**	Australia, USA	Retrospective, multicenter, clinic-based	200	77(7)	121/79	NA	2	CFP, NIR, SD-OCT
**Hogg RE, *Ophthalmology*, 2014[28]**	Italy, Portugal, UK	Prospective, multicenter, clinic-based	105	76(7.5)	52/53	Every 6 months	2	AFI, CFP, ICG, IR, RF, SD-OCT
**Chang YS, *Acta Ophthalmol*. 2015[31]**	Korean	Retrospective, single-center, clinic-based	81	75(6.1)	60/21	Every month in first 6 months, and then every 1–4 months	2	BC, CFP, IR, SD-OCT

NA = not available; AFI = auto fluorescence imaging; BC = blue channel; BLP = blue light photography; CFP = color fundus photograph; FA = fluorescein angiography; ICG = indocyanine green angiography; IR = infra-red image; NIR = near-infrared reflectance; RF = red free; SD-OCT = Spectral domain optical coherence tomography.

**Fig 2:**
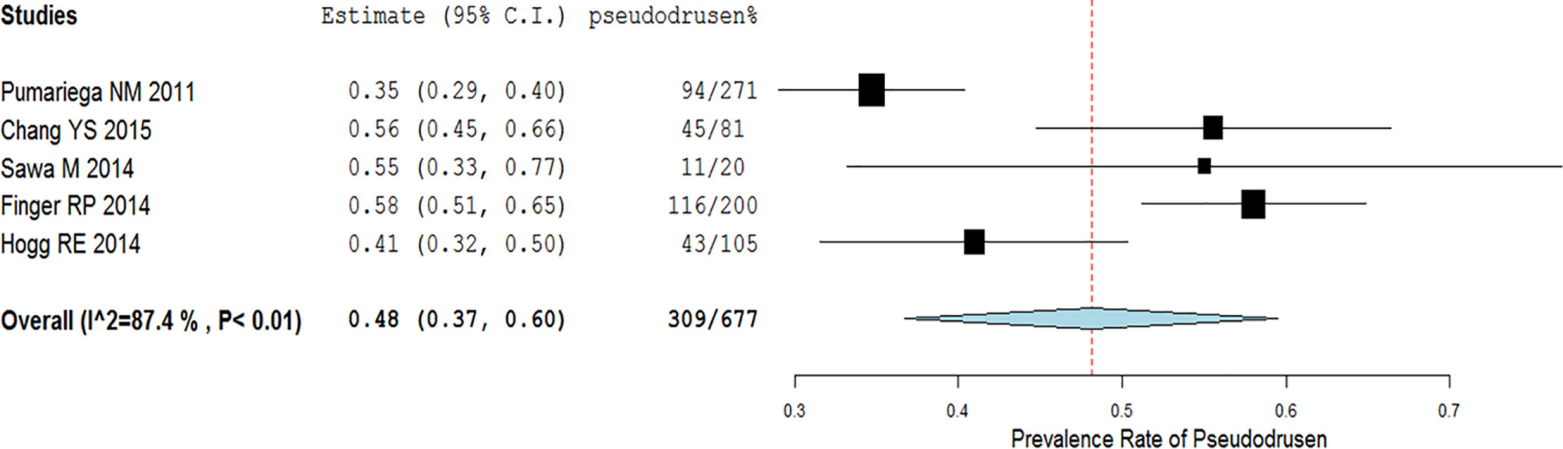
The prevalence rate of pseudodrusen in the fellow eye of patients with unilateral neovascular age-related macular degeneration.

These 5 studies used various imaging modalities to detect pseudodrusen, with the number of imaging modalities ranging from 3 to 6. Among them, 4 studies used SD-OCT,[27, 28, 31, 32] 4 studies used color fundus photographs,[27–29, 31] and 3 studies used blue light or blue channel images.[29, 31, 32] The prevalence rate at baseline of pseudodrusen in the fellow eye from the individual studies ranged from 34.7% to 58.0%. After combining these results, the pooled estimate of the prevalence rate was 48.1% (309/677; 95% Cl: 36.7–59.5%, I^2^ = 87%, Fig 2).

**Fig 3:**
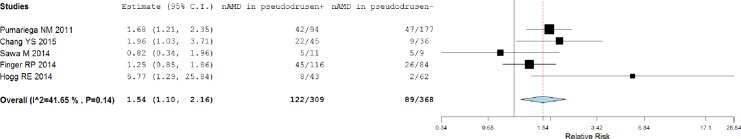
Association between pseudodrusen and incidence of the neovascular age-related macular degeneration (nAMD) in the fellow eye.

The annual incidence rate of nAMD in patients with pseudodrusen from the individual studies ranged from 9.3% to 21.2%, with a pooled annual incidence rate of 15.3% (95% Cl: 11.2–19.4%). Correspondingly, the annual incidence of nAMD in patients without pseudodrusen ranged from 6.5% to 26.8%, with a pooled annual incidence rate of 12.9% (95% Cl: 6.7%-19.1%). In the pooled analysis, nAMD occurred in 122 (39.5%) of 309 patients with pseudodrusen and in 89 (24.2%) of 368 patients without pseudodrusen, for an RR of 1.54 (95% Cl: 1.10–2.16, I^2^ = 42%, Fig 3).

**Fig 4:**
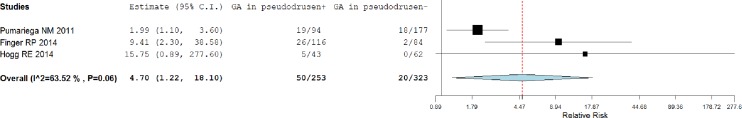
Association between pseudodrusen and incidence of geographic atrophy (GA) in the fellow eye.

Only 3 studies reported the incidence of GA.[27–29] Among these 3 studies with GA incidence (N = 576), the annual incidence rate of GA in patients with pseudodrusen ranged from 5.8% to 11.2%, for a pooled annual incidence rate of 7.9% (95% Cl: 4.7–16.8%) compared to the annual incidence rate of 2.0% (95% Cl: 0.0–4.0%) in patients without pseudodrusen. In the pooled analysis, GA occurred in 50 (19.8%) of 253 patients with pseudodrusen, and 20 (6.2%) of 323 patients without pseudodrusen, for a RR of 4.70 (95% Cl: 1.22–18.1, I^2^ = 64%, Fig 4).

**Fig 5:**
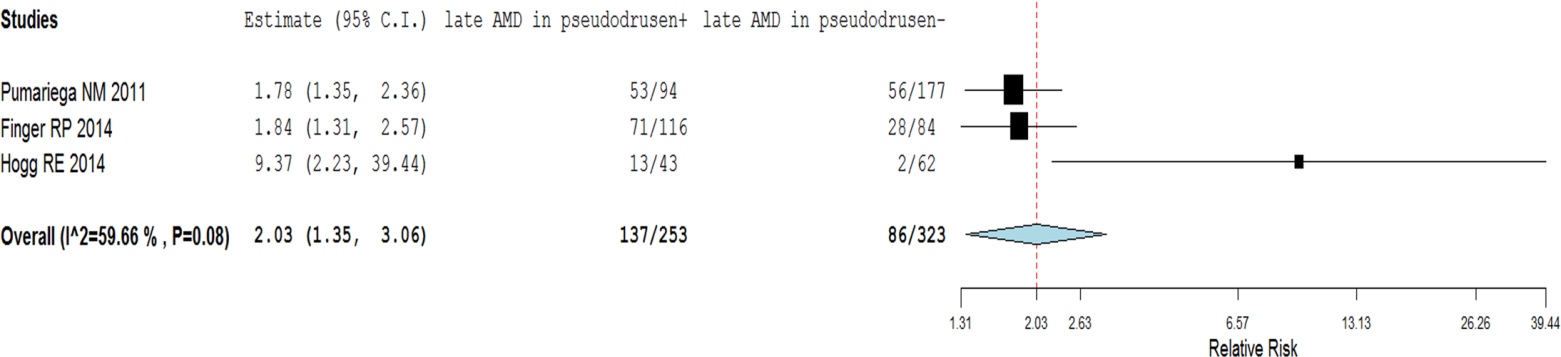
Association between pseudodrusen and incidence of late age-related macular degeneration (AMD) in the fellow eye.

Among the 3 studies that reported the incidence of both nAMD and GA,[27–29] the annual incidence rate of late AMD (either nAMD or GA) was 21.6% (95% Cl: 12.9–30.4%) in patients with pseudodrusen, and 12.9% (95% Cl: 6.7–19.1%) in patients without pseudodrusen. In the pooled analysis, late AMD occurred in 137 (54.2%) of 253 patients with pseudodrusen and 86 (26.6%) of 323 patients without pseudodrusen, for a pooled unadjusted RR of 2.03 (95% Cl: 1.35–3.06, I^2^ = 60%, Fig 5), and the covariates-adjusted (adjusted age, gender, drusen and pigmentation change) RR of 1.83 (95% Cl: 1.49–2.25, I^2^ = 33%).

To evaluate the robustness of the above results, we performed a sensitivity analysis by removing one study at a time and re-calculating the prevalence rate of pseudodrusen and the RRs for nAMD, GA, and late AMD. In these sensitivity analyses, the pooled estimates of the prevalence rate of pseudodrusen ranged from 46% to 52%, indicating a robust estimate of the pseudodrusen prevalence rate. Similarly, the pooled RR for the nAMD ranged from 1.5 to 1.7, indicating the result is not substantially influenced by any single study. However, the estimate of RR for GA was substantially influenced by the study by Pumariega et al. in that the pooled RR is 10.4 when this study is excluded, and 3.4 to 3.8 when this study is included. The pooled RR for late AMD is substantially influenced by the study of Hogg et al. in that the pooled RR for late AMD is 1.9 when this study is excluded, and approximately 3.6 when this study is included.

## Discussion

This meta-analysis evaluated the prevalence rate of pseudodrusen and its association with the incidence of nAMD and GA in the fellow eye of patients with unilateral nAMD. Our meta-analysis found that the prevalence rate of pseudodrusen in the fellow eye was high (48.1%) and that pseudodrusen was associated with an increased risk of late AMD development, with a 1.5 times higher risk of nAMD, a 4.7 times higher risk of GA, and an approximately 2 times higher risk of late AMD in the fellow eye with pseudodrusen compared to fellow eyes without pseudodrusen.

The association of pseudodrusen with nAMD and GA had similar magnitude of risk ratio as the other risk factors of late AMD such as age, smoking, large drusen and pigmentation changes.[33–35] Considering the high prevalence rate of pseudodrusen (48.1%) and its strong independent association with late AMD development, pseudodrusen should be included into the prediction model for late AMD. Although currently there is no effective therapeutics for GA, new drugs for treating or preventing GA are being tested in randomized clinical trials. Adding pseudodrusen into prediction model may help identify and follow-up high risk patients for enrollment into GA clinical trials.

The prevalence rate of pseudodrusen in the fellow eye in the included studies varied from 34.7% to 58.0%. The earliest study[29], which primarily used blue light photography without using SD-OCT to detect the presence of pseudodrusen, reported a lower prevalence rate (34.7%) than the other studies that included SD-OCT as a method of pseudodrusen detection (41 to 58%).[27, 28, 31, 32] Although this earlier study also used red free photographs and blue channel photographs to improve the pseudodrusen detection, the sensitivity of blue channel and red free images for detecting pseudodrusen is relatively low when compared with SD-OCT.[36, 37] A recent study reported that blue channel images had a sensitivity of 45.5% and a specificity of 92.0%, while SD-OCT had a sensitivity of 99.3% and a specificity of 100%.[38] The differences in imaging modalities for the detection of pseudodrusen may explain the difference in the prevalence rates of pseudodrusen across the studies. However, differences in age and gender across the studies may also partially contribute to the differences in the prevalence rates of pseudodrusen because female gender and older age are associated with a higher rate of pseudodrusen.[24] The high prevalence of pseudodrusen (48%) found in our meta-analysis indicates that pseudodrusen are not a rare phenotypic feature but rather a common phenotypic hallmark in the fellow eye of patients with unilateral nAMD.

In contrast to the detection of pseudodrusen, the determination of nAMD in the individual studies was based on fluorescein angiography and/or indocyanine green angiography. The incidence of nAMD depended on the length of follow-up. In these studies, the mean length of follow-up ranged from 2 to 4 years. To account for the differential length of follow-up, we calculated the annual incidence rate of late AMD using person year analyses. The annual incidence rate of nAMD in the fellow eyes of patients with pseudodrusen was higher than the fellow eyes without pseudodrusen (15.3% vs. 12.9%). A previous meta-analysis found the cumulative incidence rate of nAMD in the fellow eye to be 11.1% to 12.2% after 1 or 2 years, respectively[2], which is very similar to our pooled incidence of nAMD in patients without pseudodrusen and is lower than the incidence of nAMD in patients with pseudodrusen.

Five studies evaluated the association of pseudodrusen with the incidence of nAMD in the fellow eye and the reported results are quite inconsistent, with RRs ranging from 0.82 to 5.77. Our meta-analysis of these 5 studies found that pseudodrusen are statistically significantly associated with nAMD with a RR of 1.54 (95% CI: 1.10–2.16), demonstrating that pseudodrusen conferred an increased risk of nAMD development in the fellow eye of patients with unilateral nAMD. This result suggests that even among eyes at a high risk of developing AMD, the presence of pseudodrusen may exacerbate the development of nAMD. This association may be related to a thinning of the choroidal thickness in eyes with pseudodrusen,[39–43] which may result in decreased choroidal perfusion, subsequent ischemia of the choroid and retina,[5] and eventual development of neovascularization.

Three studies evaluated the association between the presence of pseudodrusen and the incidence of GA in the fellow eye of patients with unilateral neovascular AMD.[27–29] The results from these studies varied substantially. In the study with the largest sample size (N = 271) by Pumariega et al., a statistically significant association with a RR of 1.99 (95% CI: 1.1–3.6) was observed, while the study (N = 200) by Finger et al. reported a much larger and statistically significant association with a RR of 9.4 (95% CI: 2.3–38.6). The study (N = 105) by Hogg et al. reported the largest association with a RR of 15.8 (95% CI: 0.89–278), although it is not statistically significant due to the small number of fellow eyes with pseudodrusen that developed GA (n = 5), and none of the fellow eyes without pseudodrusen developed GA. Although the pooled analysis from these 3 studies showed pseudodrusen were a risk factor for the development of GA with a pooled RR of 4.70, its 95% CI is very wide, ranging from 1.2 to 18.1, and there is substantial heterogeneity across studies (I^2^ = 64%). It is uncertain whether the adjustment of well-known risk factors of GA (e.g., age, smoking) will substantially change the association because these covariates-adjusted risk ratios from individual studies are not available for calculating the pooled covariate-adjusted risk ratio for GA. Future large cohort studies are needed to validate this association. Similar to the findings from this meta-analysis, cross-sectional studies have also found that the presence of pseudodrusen was associated with the presence of GA in AMD patients.[44–46] Although the mechanism underlying the association between pseudodrusen and GA is unknown, histological studies have shown that pseudodrusen are derived from RPE cells and contain some proteins shared with drusen,[47] but the lipid composition differs distinctly from drusen.[48] The presence of either pseudodrusen or drusen in an eye may suggest a disorder in the transport mechanism of the RPE. The resulting overproduction of secretions could be inflammatory or toxic,[49] thus conversely aggravating the damage of adjacent RPE cells, prompting the death of dysfunctional RPE cells and the development of GA.

Among the 3 studies that evaluated both nAMD and GA,[27–29] the pooled RR for the development of late AMD was 2.03 (95% CI: 1.35–3.06). Adjustment by other covariates (age, gender, drusen and pigmentation change) did not substantially change the association with pooled risk ratio of 1.83 (95% Cl: 1.49–2.25). This finding is consistent with the results from both population-based studies[15–17] and clinical-based investigations.[5, 7, 9, 18–20] Although neovascularization and GA are two different forms of late stage AMD with different pathogenesis, pseudodrusen seems to be more strongly associated with GA (RR = 4.7) than nAMD (RR = 1.5). This finding is consistent with the two-compartment nature of pseudodrusen, which includes the subretinal drusenoid deposits in the subretinal space and choroid as the second compartment, because choroidal thinning has been established as a companion of pseudodrusen. The two compartment anatomy suggest that the more general term “reticular macular disease”, which is not restricted to deposits in the subretinal space. In line with this two-compartment anatomy, it has been suggested that choroidal insufficiency may even be the primary reason, and that pseudodrusen are consequent to choroidal vascular disease.[27, 44, 46, 50] Xu et al found that the most common form of GA, multilobular geographic atrophy, is highly associated with RPD and has an architecture that resembles choroidal lobules. Thus pseudodrusen and GA may be strongly related simply because they are both manifestations of the same underlying disease process.

The strength of this meta-analysis is generalizability. Our comprehensive review resulted in 5 eligible studies representing 8 countries from 4 different continents. However, the results of these studies showed a substantial amount of heterogeneity. Differences in the imaging modalities used to detect pseudodrusen, sample sizes, and lengths of follow-up likely contributed to this heterogeneity. This meta-analysis is also limited in that the pooled covariates-adjusted risk ratio for nAMD and GA could not be calculated, due to the limited covariates data from these studies. As a result, larger cohort studies are needed to validate the findings from this meta-analysis, particularly for the associations of pseudodrusen with nAMD and GA.

## Conclusions

Pseudodrusen are present in about half of the fellow eyes of patients with unilateral nAMD. The presence of pseudodrusen in the fellow eye is associated with a 1.5 times higher risk of developing nAMD, a 4.7 times higher risk of developing GA, and a 2 times higher risk of developing late AMD. Pseudodrusen should be considered as a risk factor for late AMD development.

## Supporting Information

S1 File Prisma Checklist. (DOC)

S2 File PLOSOne_Clinical_Studies_Checklist. (DOCX)

S3 File Editorial Certificate. (PDF)
